# Advances in Novel Biologics Targeting BAFF/APRIL in the Treatment of IgA Nephropathy

**DOI:** 10.3390/cells15030240

**Published:** 2026-01-26

**Authors:** Yiduo Xu, Yingqiu Mo, Youhua Xu

**Affiliations:** 1 Faculty of Chinese Medicine, State Key Laboratory of Mechanism and Quality of Chinese Medicine, Medical Sciences Division, Macau University of Science and Technology, Taipa, Macao, China; 2230022124@student.must.edu.mo (Y.X.); 2230022236@student.must.edu.mo (Y.M.); 2 Macau University of Science and Technology Zhuhai MUST Science and Technology Research Institute, Hengqin, Zhuhai 519031, China

**Keywords:** IgA nephropathy, BAFF, APRIL, targeted therapy

## Abstract

**Highlights:**

1. IgA nephropathy is a common glomerular disease and a leading cause of progression to end-stage renal disease.
2. BAFF and APRIL induce excessive B-cell activation by binding to receptors TACI, BCMA, and BAFF-R, leading to the overproduction of galactose-deficient IgA1 (Gd-IgA1).
3. Mechanism of action of BAFF/APRIL biological agents in treating IgA nephropathy.
4. Clinical trial progress of BAFF/APRIL biological agents in treating IgA nephropathy.

**Abstract:**

IgA nephropathy (IgAN) is the most common primary chronic glomerular disease worldwide. Its clinical features include proteinuria and complement pathway activation, which are the strongest predictors of progression to renal failure. This disease can occur at any age. Approximately 30–40% of IgAN patients progress to end-stage renal disease (ESRD) within 20–25 years after diagnosis, making it one of the major causes of ESRD. As understanding of the autoimmune development of IgA nephropathy (IgAN) grows, research shows that BAFF and APRIL promote B-cell activation by binding to the receptors TACI, BCMA, and BAFF-R. This results in the overproduction of galactose-deficient IgA1 (Gd-IgA1), which helps drive the progression of IgA nephropathy. B-cell and plasma cell-targeted therapies, such as biologics against BAFF/APRIL, can precisely and effectively improve patient symptoms. Corresponding agents have now been successfully developed and are administered via subcutaneous or intravenous injection. Clinical trials have demonstrated the significant effectiveness of this approach, especially in reducing proteinuria, stabilizing eGFR, and lowering Gd-IgA1 levels. Although current trial data for BAFF/APRIL-targeted biologics in IgA nephropathy are promising, these new treatments need ongoing clinical monitoring for long-term infection risks and potential drug resistance. This article focuses on the application of BAFF/APRIL biologics in the treatment of IgA nephropathy, addressing gaps in existing literature. While prior studies have emphasized the mechanisms of action of these drugs in IgA nephropathy, they have lacked a comprehensive summary of the current status of specific drug research and clinical progress.

## 1. Introduction

IgA nephropathy (IgAN), as a primary glomerular disease first proposed by Jean Berger and Hinglais in 1968, is now considered to be the most common primary glomerulonephritis worldwide and a major cause of chronic kidney disease (CKD) and renal failure [1]. According to valid statistics, the annual incidence of adult IgAN is estimated to be at least 25 cases per million population per year (pmp/year) globally, with the highest incidence in Asia accounting for 30–60% of all renal biopsies, especially in Japan and China [2,3]; and medium prevalence in Europe, 20–30% of all renal biopsies, with higher prevalence in the Nordic countries than in the Southern European countries [4].

The pathogenesis of IgAN is still not fully elucidated. Core mechanism involves the abnormal production of galactose-deficient IgA1 (Gd-IgA1) due to mucosal immune dysregulation, the formation of autoantibodies against this abnormality, the deposition of immune complexes in the renal mesangial area, complement system activation, and the resulting inflammatory response and progressive renal damage [1,5,6,7,8]; and converging mechanism will finally lead to glomerular and tubulointerstitial inflammation, renal fibrosis, and renal failure [9,10]. Clinically, IgAN commonly manifests as recurrent episodes of gross hematuria or microscopic hematuria, with the most frequent presentation being asymptomatic hematuria accompanied by varying degrees of proteinuria [11].

Current conventional therapies for IgA nephropathy mainly work through immunosuppression, anti-inflammatory effects, and hemodynamic regulation, but they do not provide disease-specific targeted treatment. However, they are often linked to serious side effects, including osteoporosis from prolonged use, infections, gastrointestinal issues, and a relatively high rate of recurrence afterward discontinuation [4]. While budesonide offers a targeted therapeutic approach for IgA nephropathy, the associated treatment costs remain substantially high [12]. In regions like Japan, tonsillectomy combined with steroid pulse therapy (TSP) is also used to remove a potential source of abnormal IgA1 production. However, this method is not yet widely recommended in international guidelines, and its long-term effectiveness remains a topic of debate [13]. Although these traditional treatment approaches can manage the condition to some degree, exploring new therapeutic methods remains essential for patients’ long-term well-being.

Recent evidence suggests pivotal role of BAFF/APRIL system in development of IgAN. Therefore, precision intervention targeting IgAN at its source represents the most prominent and novel feature of BAFF/APRIL biologics, offering an entirely new therapeutic strategy for the treatment of this disease [14]. This type of targeted therapy can precisely target the source to stop the production of pathogenic IgA1, unlike traditional treatments that only address the symptoms of IgA nephropathy. It thus provides a new treatment option for IgAN. The first biologics targeting the immunopathological mechanisms of IgAN—sepelimumab and etanercept—demonstrated significant efficacy in their respective Phase III clinical trials, namely the VISIONARY and ORIGIN studies [15,16], and BAFF/APRIL inhibitors have demonstrated stable eGFR in clinical trials; additionally, the convenient administration method greatly improves ease of dosing, which improves patients’ quality of life. Therefore, exploring the applications, mechanisms, clinical advances, and challenges of BAFF/APRIL-targeting biologics in the treatment of IgAN is important for advancing precision medicine in this disease.

## 2. Role of BAFF/APRIL Pathway in IgAN

### 2.1. BAFF/APRIL Dual Ligands Mechanism and Its Signaling Pathway

**Figure 1:**
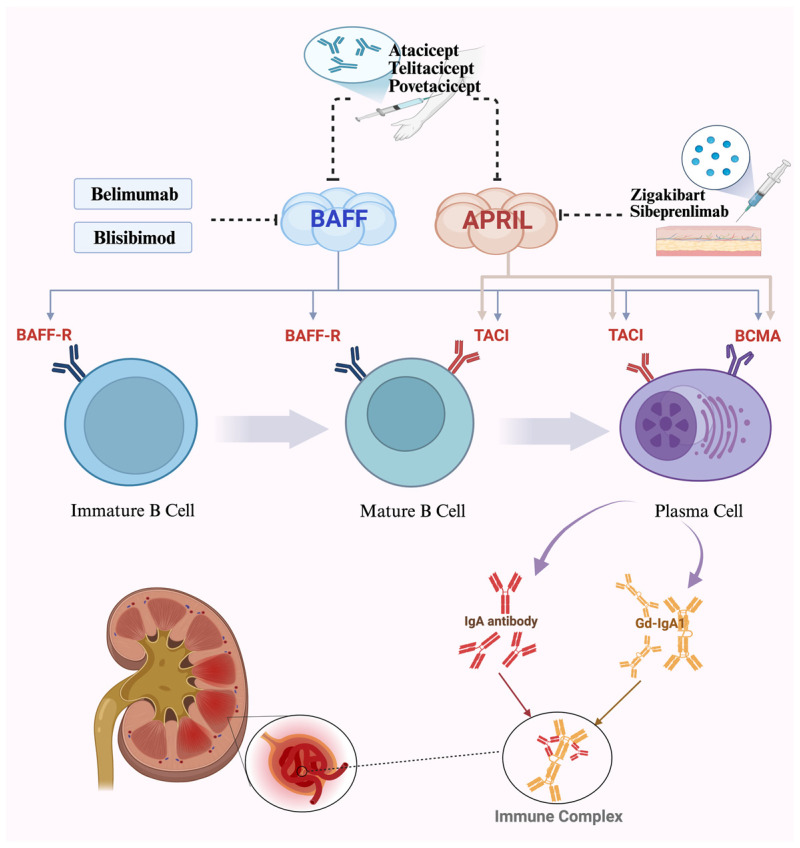
BAFF primarily binds to BAFF-R, while APRIL mainly binds to TACI and BCMA receptors. By influencing B-cell differentiation and plasma cell survival, BAFF and APRIL induce the secretion of Igd-IgA1 (antigen) and anti-Gd-IgA1 autoantibodies, which ultimately form pathogenic immune complexes. These complexes deposit in the renal mesangial region, triggering inflammatory responses in the kidneys. Created in BioRender. Xu, Y. https://BioRender.com/2zyp2i4, accessed on 5 January 2026.

B lymphocytes activating factor (BAFF) and apical proliferation-inducing ligand (APRIL) within the tumor necrosis factor (TNF) family are structurally similar yet functionally distinct members of the TNF superfamily [17]. Although these two receptors have distinct functions, they are closely interconnected. BAFF is typically processed on the cell surface. Experimental evidence indicates that excessive BAFF production in the body can trigger severe autoimmune diseases in mice: systemic lupus erythematosus (SLE) and Sjögren’s syndrome (SS). In contrast, APRIL undergoes processing intracellularly via prohormone convertase and functions exclusively as a soluble factor, promoting proliferation in both normal and tumor cell lines [18,19]. BAFF specifically binds to BAFF receptors (BAFF-R), TACI, and BCMA, while APRIL primarily interacts with BCMA and TACI but not BAFF-R [20,21]. In autoimmune diseases, BAFF and APRIL can form heterotrimers through noncovalent interactions [22]. As crucial factors for B lymphocytes survival, maturation, and differentiation into plasma cells, they jointly regulate B lymphocytes homeostasis through interactions with receptors BAFF-R, TACI, and BCMA, exerting significant influence on T-cell-independent immune responses and immunoglobulin class switching, particularly IgA [22,23]. BAFF and APRIL form complex signaling networks through shared co-receptors TACI (Transmembrane Activator and Calcium-modulating Cyclophilin Ligand Interactor) and BCMA (B-Cell Maturation Antigen) by utilizing common signaling pathways [24,25]. Experimental evidence confirms that the two cytokines BAFF and APRIL can combine with the receptor TACI, jointly activating downstream signaling pathways (such as NF-κB) and collectively promoting B lymphocytes activation and pathogenic IgA production [26,27]. In animal models, blocking the BAFF/APRIL system (such as using the TACI-Fc fusion protein) significantly reduces kidney injury and proteinuria [22]. Additionally, BAFF activates the PI3K-Akt signaling pathway through its receptor BAFF-R, thereby participating in the regulation of T-cell activation and proliferation to exert its immunomodulatory effects [28]. It has been shown that BAFF/APRIL overexpression promotes the development of IgAN by facilitating B lymphocytes differentiation, maturation, and aberrant secretion of plasma cell antibodies (Figure 1) [29].

### 2.2. BAFF/APRIL Abnormal Activation Induces Pathogenic IgA1 Production

IgAN is an autoimmune kidney disease, and B-cells play a key role in its development and progression. B-cells differentiate into plasma cells to secrete antibodies; these antibodies bind to kidney antigens either specifically or nonspecifically, ultimately forming immune complexes that cause kidney damage [30]. Specifically, B-cells, as precursors and regulatory units, are activated upon stimulation by the receptors BAFF and APRIL, undergo class-switch recombination (CSR), and ultimately differentiate into IgA-secreting B-cells [31,32]. Subsequently, these B-cells further differentiate into effector units—plasma cells (including short-lived plasmocytes and long-lived plasma cells), with the latter migrating into the bone marrow or inflammatory tissues (such as the kidneys) [33]. A pivotal antibody secreted by these plasma cells is galactose-deficient IgA1 (Gd-IgA1). Current understanding suggests a close relationship between the production of galactose-deficient IgA1 (Gd-IgA1) and the development of IgAN [34].

BAFF/APRIL over-activation plays a central role in the production of Gd-IgA1. Studies have shown that elevated levels of BAFF and APRIL in patient serum are closely associated with disease severity indicators, such as proteinuria, decreased eGFR, and pathological damage [35,36]. Transcriptome analysis revealed widespread gene expression abnormalities in B-cells from IgAN patients, particularly in genes involved in B-cell activation and antibody production [33]. BAFF is essential for B-cell maturation and survival mainly through interaction with the BAFF receptor. Recent findings suggest that inhibiting BAFF significantly reduces the overall serum levels of IgA, IgG, and IgM. This indicates that BAFF inhibition does not specifically target IgA but instead has a broad regulatory effect on B-cell survival and differentiation, impacting multiple immunoglobulin classes. APRIL mainly influences B-cell differentiation and plasma cell survival through the TACI and BCMA receptors. These processes lead to increased production of IgA, particularly gammaglobulin-deficient IgA1. Experimental results demonstrate that APRIL is elevated in IgAN patients and correlates with disease severity [37]. In animal models, APRIL-neutralizing antibodies significantly reduce levels of abnormally glycosylated IgA, diminish glomerular IgA deposition, and improve proteinuria, indicating that APRIL specifically promotes the production of abnormally glycosylated IgA [38]. APRIL mainly helps keep mature B-cells alive, like long-lived plasma cells, which may be an important cellular source for ongoing production of abnormally glycosylated IgA. The initial B-cells are activated, differentiate into short-lived plasma cells (SLPC), and then mature into long-lived plasma cells (LLPC) within the bone marrow [39]. BAFF primarily targets mature B-cells and early plasma cells, with only limited direct support for long-lived plasma cells; APRIL is crucial for maintaining the survival of long-lived plasma cells, especially intestinal IgA^+^ plasma cells and bone marrow plasma cells [25]. In summary, both anti-BAFF antibodies and anti-APRIL antibodies can decrease serum IgA levels, but only anti-APRIL antibodies can block the production of abnormal antibodies and inhibit the synthesis of abnormally glycosylated IgA.

**Figure 2:**
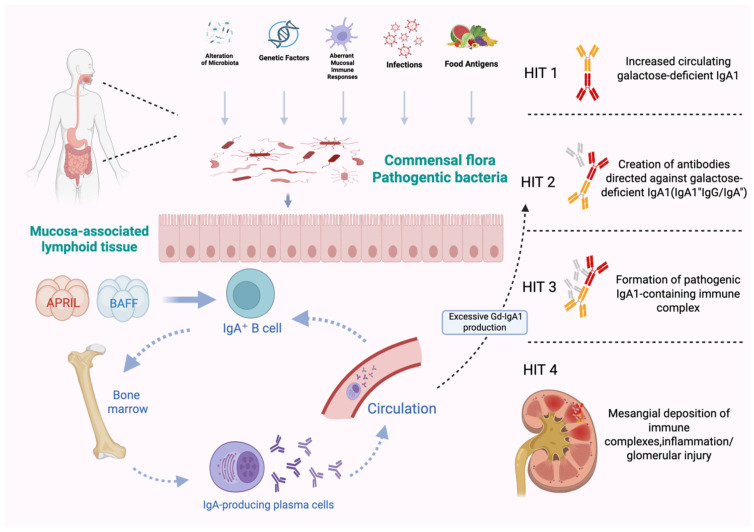
BAFF and APRIL stimulation promote B-cell differentiation and proliferation. IgA+ B-cells migrate through the lymphatic system and systemic circulation to mucosal effector sites under the regulation of homing receptors. Subsequently, they undergo hyperactivation, class switching, and differentiate into plasma cells, leading to the overproduction of galactose-deficient IgA1 (Gd-IgA1). Gd-IgA1 then forms immune complexes with autoantibodies, deposits in the glomerular mesangial region, activates the complement system, and causes inflammatory damage. Created in BioRender. Xu, Y. https://BioRender.com/39uuyhv, accessed on 16 January 2026.

Specifically, abnormally high levels of BAFF and APRIL are associated with mucosal immune dysregulation, such as in the tonsils or gut-associated lymphoid tissue. These factors stimulate IgA1 secretion by enhancing B-cell signaling pathways (e.g., the TACI receptor-mediated pathway) and promote abnormal glycosylation patterns of Gd-IgA1 (O-glycosylation defects) by regulating the expression of key glycosyltransferases [40,41,42]. High levels of BAFF and APRIL are often connected to disruptions in mucosal immunity, particularly in the gut-associated lymphoid tissue (GALT), which plays a crucial role in the development of IgAN (Figure 2) [43]. The production of IgA+ B-cells occurs within MALT through either T-cell-dependent (TCD) or T-cell-independent (TCI) pathways. The TCD pathway is initiated by B-cell activating factor (BAFF or BLyS) and proliferin-associated receptor-activating ligand (APRIL). After forming IgA+ B-cells, they migrate through the lymphatic system and systemic circulation to mucosal effector sites under the control of homing receptors [31]. The resulting antibodies form IgA dimers composed of two IgA molecules linked by connecting chains, and are frequently detected within renal tissue [44,45].

In short, these two cytokines, BAFF and APRIL, drive excessive and abnormal immune complex formation by Gd-IgA1 through enhanced B-cell-mediated immune responses, ultimately leading to glomerular inflammation [46,47,48].

## 3. The Shift from Conventional Therapy to Precision Therapy

The current mainstream treatment for IgAN still primarily relies on glucocorticoids and broad-spectrum immunosuppressant [49]. However, these traditional treatment regimens often demonstrate notable disadvantages. The TESTING trial indicated that CS therapy for patients was associated with severe infections [50]. While other medications also posed risks of infection, gastrointestinal disturbances, and significant financial burden, thereby exacerbating patients’ physical suffering and economic strain (Table 1) [51].

In renal protection, glucocorticoids with RAS blockers suppress immune injury, while SGLT2 inhibitors delay chronic complications via metabolic regulation [52]. Tacrolimus offers renal protection by stabilizing podocytes and reducing proteinuria [53]. Etiological treatment targets mucosal immune irregularities: tonsillectomy removes abnormal immune foci, while targeted release of budesonide specifically decreases pathogenic IgA1 production [54,55]. Tacrolimus and corticosteroids also have immunomodulatory effects. Current treatment trends emphasize developing personalized regimens based on pathological subtypes, such as prioritizing immunosuppression for active lesions and focusing on metabolic regulation for chronic lesions. This approach simultaneously achieves causal intervention and renal function preservation, thereby delaying disease progression.

Calcineurin inhibitors have been studied for a long time but have not been recommended as a treatment for IgAN. Calcineurin inhibitors (CNIs) used in managing IgA nephropathy are typically classified as immunosuppressive agents, with tacrolimus and cyclosporine representing this category [56]. Previous studies have established that calcineurin inhibitors can impede the production of interleukin-2 and its receptor by restricting the dephosphorylation of activated T-cell nuclear factors. They directly suppress T-cell activity and potentially macrophage activity, while exerting an indirect inhibitory effect on B-cells [57,58]. While both BAFF and APRIL biologics focus on B-cells in treating IgA nephropathy, calcineurin inhibitors work more indirectly, only gently reducing B-cell activity instead of directly targeting them. Plus, their way of working is quite broad and does not specifically involve the BAFF/APRIL pathway.

Glucocorticoids primarily mitigate inflammatory responses within the glomeruli and renal tubulointerstitium by inhibiting the release of various inflammatory mediators, such as TNF-α and IL-6 [50]. However, they are unable to diminish the production of Gd-IgA1 [59]. RAS inhibitors primarily mitigate IgA nephropathy by decreasing proteinuria via hemodynamic mechanisms that enhance intraglomerular function pressure [60]. Sodium–Glucose Transporter 2 Inhibition (SGLT2) mainly improves hypoxia in IgA nephropathy by helping to lower glomerular capillary pressure and reduce renal oxygen consumption [61] Nefecon treatment for IgA nephropathy constitutes a significant advancement in recent years. It not only demonstrates a high affinity for glucocorticoid receptors but also specifically modulates mucosal immunity, thereby reducing pathogenic IgA1 production at its source. However, unlike BAFF/APRIL biologic therapies targeting IgA nephropathy, Nefecon demonstrates a positive effect on BAFF biomarkers, whereas its impact on APRIL biomarkers remain to be determined [62]. While it targets B-cells, it does not address IgA nephropathy through the BAFF/APRIL pathway. With the elucidation of the BAFF/APRIL pathway’s pivotal role in pathogenic IgA1 production, the advantages of BAFF/APRIL biologics in precision therapy for IgAN have become increasingly evident (Table 2) [33,63]. Abundant evidence demonstrated that this type of targeted intervention strategy not only reduces pathogenic IgA1 levels but also avoids the broad range of side effects associated with conventional treatment regimens [33,64,65].

**Table 1:** Conventional strategy for IgAN.

Treatment Plan	Medication	Mechanism of Action	Adverse Reactions	Primary Endpoint	Trial Status	References
Glucocorticoids	Prednisone, Prednisolone	By suppressing immune inflammatory responses, reducing IgA deposition in the mesangial region, and inhibiting complement activation, it alleviates proteinuria and delays the deterioration of renal function.	Long-term use may lead to serious adverse reactions, such as osteoporosis, diabetes, and increased risk of infection.	The first occurrence of a sustained 40% decrease in eGFR, the development of kidney failure, or death due to kidney disease.	Completed (NCT01560052)	[50]
Immunosuppressants	Cyclophosphamide, Mycophenolate Mofetil	Reduce immune complex deposition by inhibiting T-cell and B-cell proliferation or antibody production.	Cyclophosphamide may cause bone marrow suppression and gonadal toxicity, while mycophenolate may lead to gastrointestinal symptoms and an increased risk of infection.	A composite of ≥40% decrease in eGFR from the baseline, onset of kidney failure and all-cause mortality.	Completed (NCT01854814)	[66,67,68]
Angiotensin-Converting Enzyme Inhibitor (ACEI)/Angiotensin II Receptor Antagonists/Blockers (ARBs)	RAS inhibitor	For critically ill patients, to synergistically control immune responses and hemodynamic abnormalities	The safety of ACEI/ARB therapies is well established	Complete remission (CR) of proteinuria at 12 months	N/A	[69,70]
Tonsillectomy with Steroid Pulse Therapy (TSP)	/	The tonsils may participate in IgAN pathogenesis through a phosphocholine (PC)-specific immune response. Tonsillectomy removes the potential source of abnormal IgA1 production.	Bleeding and infection. Tonsillectomy with Steroid Pulse Therapy (TSP) is widely used in Japan, but international guidelines have not yet universally.	A decline in kidney function with a 50% increase in sCr from baseline or ESKD with dialysis initiation and preemptive kidney transplantation.	N/A	[55,66,71]
Sodium–Glucose Transporter 2 Inhibition (SGLT2)	Dapagliflozin, mpagliflozin and canagliflozin	By reducing intraglomerular pressure, improved tubuloglomerular feedback, and attenuation of renal inflammation and fibrosis.	Genitourinary infections and volume depletion, but such risks are manageable with appropriate monitoring.	Between-arms difference in proteinuria reduction within 6 months in ACIgAN and within 12 months in CHRONIgAN study.	Completed (NCT04662723)	[52,72]
Calcineurin inhibitors (CNI)	tacrolimus	By binding T lymphocyte-specific FK506-binding protein to form the TAC–FKBP12 complex, which then binds to calcineurin and alters the transcription of numerous genes in T-cells, thus lowering the immunological response.	Gastrointestinal discomforts, headache, tremor, and coldness of extremities.	It is defined as the percent change (%) of final UACR (pcUACR) compared to the baseline value.	Completed (NCT1224028)	[73]
Budesonide	Nefecon	To deliver a local potent anti-inflammatory effect in the distal ileum and proximal colon.	Hypertension, peripheral edema, muscle spasms, acne, dermatitis, weight gain, dyspnea, facial edema, dyspepsia, fatigue, and excess hair.	Time-weighted average of eGFR over 2 years.	Completed (NCT03643965)	[74]

Note: ACEI, angiotensin-converting enzyme inhibitor; ARB, angiotensin receptor blocker; TSP, Tonsillectomy with Steroid Pulse Therapy; PC; phosphocholine; SGLT2, Sodium–Glucose Transporter 2 Inhibition; CNI, Calcineurin inhibitors.

## 4. BAFF/APRIL Therapy for IgAN

### 4.1. Single-Targeted BAFF Inhibitor

#### 4.1.1. Belimumab

Belimumab is a recombinant human IgG-1λ monoclonal antibody that exerts its effects by specifically inhibiting B-cell activating factor (BAFF) [75]. The U.S. FDA approved belimumab for the treatment of systemic lupus erythematosus (SLE) in 2011, marking the first SLE treatment approved in nearly 60 years [76]. In a case report of a pediatric patient with refractory IgAN, the addition of belimumab to conventional therapy (such as immunosuppressive regimens) resulted in renal response and reduced proteinuria [77]. Aside from isolated clinical case reports, there are no other studies or reports on using this drug to treat IgA nephropathy.

#### 4.1.2. Blisibimod

Blisibimod is a targeted BAFF inhibitor studied in patients with systemic lupus erythematosus. It was developed based on the success of belimumab, aiming to target both soluble and membrane-bound BAFF. Because of its high affinity, it effectively blocks all three forms of BAFF (soluble BAFF trimer, soluble 60-mer, and membrane BAFF) [78]. The Phase II/III clinical trial of Blisibimod (BRIGHT-SC; NCT02062684) was initiated in 2017 and has now concluded, although the results of the trial remain unpublished. Existing evidence suggests that the drug reduces proteinuria levels in patients with IgA nephropathy, exhibiting superior efficacy compared to placebo. However, the Phase III trial of this medication (BRILLIANT-SC; NCT02052219), which was initiated in 2015, was terminated prematurely [70].

Compared to monotherapy with BAFF antibodies, current research on BAFF/APRIL biologics for treating IgA nephropathy has shifted its focus toward both monotherapy with APRIL antibodies and dual inhibition of BAFF and APRIL antibodies.

### 4.2. Single-Targeted APRIL Inhibitor

#### 4.2.1. Sibeprenlimab

It is a humanized IgG2 monoclonal antibody that specifically binds to APRIL, blocking its biological activity to reduce the production of pathogenic IgA (such as gammaglobulin deficiency-like IgA1) and decrease the deposition of IgA-related immune complexes in the kidneys, ultimately alleviating renal injury [79,80,81]. Its most distinctive feature is direct intervention at the cause of the disease, enabling precise treatment from the source rather than merely alleviating symptoms [35]. This offers a novel precision treatment approach for IgAN. A monthly subcutaneous injection is one of the advantages of Sibeprenlimab administration. Currently, Sibeprenlimab has entered Phase III clinical trials. During this phase, Sibeprenlimab has demonstrated efficacy: significantly reducing proteinuria, stabilizing renal function, and exhibiting favorable safety [80,82]. However, it remains in the mid-to-late stages of development and requires further data to support its routine clinical application.

#### 4.2.2. Zigakibart

It is a humanized IgG4 monoclonal antibody that inhibits interactions between APRIL and its receptors BCMA and TACI, thereby reducing B-cell activation and abnormal IgA production [25,35,83]. The mode of administration for Zigakibart is a monthly subcutaneous injection. Compared to therapies requiring intravenous infusion, this treatment offers patients greater convenience and significantly improves treatment adherence. Zigakibart significantly reduces levels of free APRIL, IgA, and IgM, moderately lowers IgG levels, and most importantly, specifically reduces the production of galactosodeficiency IgA1 (Gd-IgA1), a key pathogenic factor in IgAN [84,85]. Clinical trials demonstrated that after treatment with Zigakibart, patients experienced a 60% reduction in proteinuria, with estimated glomerular filtration rate (eGFR) gradually stabilizing. All subjects exhibited good tolerability, and no serious adverse reaction was reported [85]. Phase II clinical data have confirmed the safety and efficacy of Zigakibart, with Phase III clinical trials currently underway, offering a new targeted treatment option for IgAN.

### 4.3. Dual Inhibitor Targeting Both BAFF and APRIL

#### 4.3.1. Atacicept

It is an innovative TACI-Fc fusion protein whose molecular structure comprises the extracellular domain of the transmembrane activator and calmodulin-binding ligand (TACI) fused to the human IgG1 Fc segment [32,86]. Atacicept is typically administered as a once-weekly subcutaneous injection. By inhibiting the activity of these two cytokines, it reduces the production of pathogenic IgA1 and immune complex formation, thereby improving proteinuria and stabilizing renal function [37,86,87]. Double-blind studies have demonstrated that Atacicept (in both the 150 mg and 75 mg dose groups) significantly reduced the urine protein-to-creatinine ratio (UPCR) at 24 weeks of treatment, indicating its favorable therapeutic efficacy [88]. Another Phase III clinical trial (ORIGIN) confirmed the safety and pharmacodynamic value of Atacicept [16]. Atacicept shows promise as a novel targeted therapy in the treatment of IgAN.

#### 4.3.2. Telitacicept

It is a dual-target fusion protein targeting both B-lymphocyte stimulator (BLyS) and apical protein-related inducible ligand (APRIL) [89]. Telitacicept is administered as a once-weekly dose via subcutaneous injection. Telitacicept achieves this by inhibiting the activity of these two cytokines, thereby reducing levels of Gd-IgA1 and IgA-containing immune complexes, significantly alleviating proteinuria in patients [90,91]. It can also alleviate local inflammatory responses in the glomeruli by reducing IgA deposition and the formation of immune complexes, thereby minimizing further renal damage [92,93,94]. Reports indicate that Telitacicept combined with corticosteroids and mycophenolate mofetil (MMF) demonstrated favorable safety and efficacy in the induction therapy of IgAN, effectively preserving patients’ renal function [95,96,97]; additionally, Telitacicept has demonstrated potential therapeutic value in refractory IgAN and recurrent IgAN (such as recurrence after kidney transplantation) [98]. Currently, Telitacicept has demonstrated promising therapeutic potential not only in multiple clinical studies for IgAN but also in clinical trials for other autoimmune diseases, such as systemic lupus erythematosus (SLE) and multiple sclerosis (MS) [99,100]. Telitacicept was approved in China in 2021 for the treatment of systemic lupus erythematosus (SLE). In March 2024, it received approval from China’s National Medical Products Administration (NMPA) for the treatment of IgAN.

#### 4.3.3. Povetacicept

Povetacicept is an engineered ‘TACI-Fc’ fusion protein, with the TACI-Fc variant created through directed evolution that shows improved binding to both APRIL and BAFF [101]. TACI is a B-cell surface receptor capable of concurrently binding BLyS and APRIL with high affinity. By inhibiting BAFF and APRIL, this pharmaceutical agent prevents the synthesis of pathogenic gammaglobulin deficiency-associated IgA1 (Gd-IgA1), thereby mitigating renal damage resulting from immune complex deposition and exerting a disease-modifying effect [102]. In the 2024 RUBY-3 povetacicept (NCT05732402) study phase, at 9 months (n = 6), treatment with 80 mg povetacicept resulted in a 64% reduction in urinary protein-to-creatinine ratio (UPCR) and a 69% decrease in Gd-IgA1 protein; 67% of patients achieved remission status, with all patients exhibiting hematuria at baseline experiencing resolution of this symptom. Renal function remained stable, demonstrating significant clinical efficacy [103]. In late-stage RUBY-3 trials, povetacicept (NCT05732402) showed that IgAN patients that received the 80 mg dose achieved a 64% mean reduction from baseline in 24-h urine protein-to-creatinine ratio (UPCR), and estimated glomerular filtration rate (eGFR) remained stable at week 48. Approximately 90% of patients experienced resolution of hematuria, with 53% achieving clinical remission. The 240 mg dose group demonstrated efficacy comparable to the 80 mg [104].

Based on the previous discussion, we classify the patient subgroups appropriate for APRIL inhibitor therapy into the following three groups: 1. Patients mainly rely on APRIL for plasma cell survival—APRIL primarily maintains long-lived plasma cells through the TACI and BCMA receptors, while BAFF has a weaker role in supporting these cells. 2. Patients at higher risk of infection, including children, elderly individuals, those with chronic infections (such as recurrent respiratory or gastrointestinal infections), or those with immunocompromised conditions, may be prioritized for APRIL selective inhibitors to maintain some BAFF-mediated immune defense functions. 3. Patients aiming to minimize the impact of treatment on the overall immune system and focus intervention solely on the pathogenic IgA1 pathway—APRIL specifically promotes the production of abnormally glycosylated IgA1, while BAFF exerts regulatory effects across multiple immunoglobulin classes.

Monotherapy with anti-APRIL may be sufficiently effective for most IgAN patients, although dual BAFF/APRIL blockade offers greater mechanistic potential, especially for patient groups whose plasma cell survival relies on dual BAFF/APRIL signaling. Future studies should further examine the long-term efficacy and safety of these two treatment options.

**Table 2:** BAFF/APRIL Biological Agents for the Treatment of IgA Nephropathy.

Medication	Mechanism of Action	Research Progress	Duration of Treatment	Proteinuria	Effect on eGFR	Side Effects	Reference
Belimumab	Belimumab inhibits the proliferation and survival of overactive B-cells by binding to BAFF, thereby reducing the production of pathogenic IgA1.	Existing literature does not provide specific clinical conversion rate data for belimumab in immune-mediated glomerulonephritis with large globular deposits (IgAN), with only isolated case reports available.	N/A	N/A	N/A	N/A	[77]
Blisibimod	Because of its high affinity, by aiming to target both soluble and membrane-bound BAFF, also effectively blocks all three forms of BAFF.	The Phase II/III clinical trial (NCT02062684) suggests it reduced proteinuria in patients with IgA nephropathy compared to placebo.	At least 60 weeks of treatment and up to 2 years.	Achievement of proteinuria ≤1.0 g/24 h OR a 50% reduction from baseline at two consecutive visits.	It improved individual rates of change in eGFR, with an annualized improvement of +6.2 mL/min/1.73 m^2^ per year.	Upper respiratory infection, nasopharyngitis, and injection site reactions.	[105]
Sibeprenlimab	A humanized IgG2 monoclonal antibody that specifically binds to and neutralizes APRIL, thereby blocking APRIL-mediated pathological processes. By inhibiting APRIL reduces the production of galactosodeficient IgA1.	TOtsuka Pharmaceutical announced interim results from the Phase III VISIONARY study (NCT05248646) for the treatment of adult IgAN. It was granted accelerated approval by the FDA.	12 months and follow-up continued until month 16.	The geometric mean ratio reductions in the 24-h urinary protein-to-creatinine ratio were 47.2 ± 8.2%, 58.8 ± 6.1%, and 62.0 ± 5.7% in the three groups, which were significantly higher than the placebo group.	At month 12, the least-squares mean changes in eGFR were −2.7 ± 1.8, 0.2 ± 1.7, and −1.5 ± 1.8 mL/min/1.73 m^2^ in the three groups, respectively, all superior to the placebo group.	The incidence of adverse events in the pooled sibeprenlimab groups was similar to that in the placebo group, with most being mild to moderate.	[15,80]
Zigakibart	A humanized IgG4 monoclonal antibody that reduces free APRIL levels by inhibiting APRIL activity, blocking its stimulatory effect on B-cells, and decreasing IgA and IgM production.	Phase I/II studies have been completed, and the Phase III beyond study (NCT05852938) clinical trial is currently underway.	The maximum treatment duration was up to 124 weeks, with core data reported at 100 weeks.	It showed a rapid and durable downward trend in proteinuria. The 24-h UPCR decreased by 60.4% compared with the baseline.	eGFR remained sustained during treatment. At week 100, the annualized eGFR slope was +0.48 ± 1.7 mL/min/1.73 m^2^.	Well-tolerated, with no treatment-emergent adverse events (TEAEs) leading to treatment discontinuation or death.	[85]
Atacicept	A novel dual-targeted fusion protein simultaneously inhibits BAFF and APRIL; by suppressing the BAFF/APRIL pathway, it significantly reduces Gd-IgA1 production.	The Phase II clinical trial of Atacicept (NCT04716231) yielded highly positive results and is currently advancing or scheduled to advance to Phase III clinical trials.	For 36 weeks. After that, an open-label extension phase was entered for 60 weeks; after the extension treatment, an additional 26-weeks of follow-up was conducted.	The UPCR decreased by 31% from baseline, representing a relative reduction of 25% compared with the placebo group.	At week 36, the eGFR in the combined 150 mg + 75 mg group increased by 1% from the baseline. Both the 150 mg and 75 mg groups achieved eGFR stabilization.	The incidence of TEAEs in the atacicept group was slightly lower than that in the placebo group, with no treatment-related deaths reported.	[16,88]
Telitacicept	By inhibiting the BLyS/APRIL signaling pathway, it reduces the production of Gd-IgA1 and its autoantibodies, thereby decreasing IgA deposition in the glomerular mesangial region.	The results of the Phase II (NCT04291781) trial conducted in China were highly positive. The international multicenter Phase III trial (NCT04291781) has now commenced.	Patients first underwent a 4-week run-in period, then were randomly assigned for 24 weeks; an additional 28-day follow-up was conducted after treatment.	At week 24, the 24-h proteinuria in the high group decreased by 49% from the baseline, with a statistically significant difference compared with the placebo group; the mean baseline 24-h proteinuria of patients was 1.86 g/d.	Both dose groups could maintain stable renal function. At week 24, the eGFR in the 240 mg group and 160 mg group increased by 2.34 mL/min/1.73 m^2^ and 4.32 mL/min/1.73 m^2^ from the baseline, respectively. The mean baseline eGFR of patients was 79.38 mL/min/1.73 m^2^.	Overall safety was favorable, with no treatment-related deaths, kidney failure, or end-stage renal disease reported; incidence of adverse events mostly mild to moderate.	[92,106,107]
Povetacicept	By inhibiting BAFF and APRIL, the drug can block the production of pathogenic galactose-deficient IgA1 (Gd-IgA1) and reduce kidney injury caused by immune complex deposition, exerting a disease-modifying effect.	Currently in the phase 1b/2a RUBY-3 (NCT05732402) study stage, key data have been released, showing significant clinical benefits.	For a 24-week period. Eligible participants had the option to participate in additional 28-week and 52-week extensions for up to a total of 104 weeks of treatment.	The 80 mg group achieved a 64.1% reduction in UPCR from baseline at 36 weeks. Another showed similar improvements in proteinuria to the 80 mg group. 67% of participants achieved remission criteria.	eGFR assessment in the two groups showed stable renal function. A sustained improvement of eGFR of + 3.3 mL/min per 1.73 m^2^.	Well-tolerated with no reports of serious safety risks. No instances of IgG < 3 g/L and no severe infections occurred.	[103,104]

Note: BAFF, B-cell Activating Factor; BLyS, B Lymphocyte Stimulator; APRIL, A Proliferation-Inducing Ligand; Gd-IgA1, galactose-deficient IgA1; TEAEs, Treatment-Emergent Adverse Events.

## 5. Current Challenges and Future Directions

### 5.1. Long-Term Safety Monitoring Requirements (Infection Risk and Immune Surveillance)

Since BAFF and APRIL are key regulators of B-cell differentiation, survival, and antibody production, BAFF/APRIL inhibitors (such as sibeprenlimab and atacicept) target these B-cell activation factors to suppress plasma cell survival and antibody production, thereby achieving therapeutic effects in IgAN [25,108,109]. However, the most direct risk of long-term suppression of B-cell function is a decline in the body’s ability to defend against pathogens, particularly extracellular bacteria and viruses [41]. Additionally, BAFF/APRIL participates in intestinal IgA class switching, so inhibiting BAFF/APRIL may also compromise the intestinal immune barrier function. Long-term use requires monitoring for respiratory or intestinal infections associated with mucosal immunity [110]. Additionally, since the function of B-cells and T-cells harboring virus-specific memory may be disrupted by BAFF/APRIL inhibitors, there is a risk of reactivation of latent varicella-zoster virus in the body [111,112,113]. Therefore, monitoring skin pain, itching, or herpes manifestations is crucial in the treatment of IgAN. Inhibition of BAFF/APRIL may lead to decreased levels of non-IgA immunoglobulins (such as IgG and IgM), so hypo-gammaglobulinemia and the associated risk of infection should also be included in monitoring parameters [114]. In response to the aforementioned risks, ongoing clinical trials of BAFF/APRIL biological inhibitors should be expedited to Phase III while simultaneously extending patient follow-up periods to promptly evaluate delayed adverse events [70]. It is recommended to incorporate BAFF/APRIL levels, Gd-IgA1 titers, and complement activation markers to correlate their efficacy and safety [35,115]. Finally, individualized monitoring plans should be developed for children, the elderly, or patients with chronic infections to ensure medication safety [116,117].

### 5.2. Mechanisms of Drug Resistance and Second Treatment Options

To date, no specific molecular mechanisms underlying its drug resistance have been explicitly reported in the existing literature. However, based on relevant studies, the following four aspects can be summarized:

(1) Activation of the TRAF6/NF-κB signaling pathway promotes the expression of fibrotic factors in glomerular mesangial cells, whereas the BAFF-R Fc chimeric protein inhibits this pathway [118,119]. If compensatory activation of alternative signaling pathways (such as other TNF family members) occurs, resistance may develop.

(2) BAFF/APRIL functional redundancy: BAFF and APRIL exhibit partially overlapping functions in B-cell activation and IgA production [108,120]. Therefore, in the treatment of IgAN, inhibiting a single molecule may lead to compensatory upregulation of BAFF.

(3) Receptor competition and heterogeneity: BAFF and APRIL share the receptor TACI, but BAFF can also exert effects through BAFF-R [48,83,121]. So when using only APRIL inhibitors to treat IgAN, therapeutic efficacy might be limited by the failure to sequester BAFF-R-mediated signals [122].

(4) Gut-kidney axis escape: BAFF/APRIL produced locally in tissues, such as the intestine, may not be affected by systemic inhibition, thereby leading to drug resistance [65,123,124].

To prevent the development of the aforementioned drug resistance, we can promptly adjust the treatment regimen when using BAFF/APRIL therapy for IgAN to achieve optimal therapeutic outcomes. In treatment, dual-target inhibitors or combination therapy with anti-BAFF/APRIL antibodies may represent a more comprehensive strategy [125,126]. Targeting downstream signaling molecules should not be overlooked; inhibiting the TRAF6/NF-κB pathway may enhance therapeutic efficacy [127,128]. Additionally, it can be used in combination with growth hormone inhibitors to enhance resistance [70]. Finally, to address escape via the gut-kidney axis, active modulation of gut immunity can reduce the intestinal sources of BAFF/APRIL [129,130].

## 6. Discussion

The TESTING trial (The Efficacy of Steroids in IgA Nephropathy Globally) was conducted among Chinese participants with a high disease prevalence. The trial was prematurely halted in the oral, high-dose, daily methylprednisolone group due to a high number of serious adverse events. After restarting the trial, administering a lower steroid dose on alternate days, combined with increased prophylaxis against pneumocystis pneumonia, has reduced infection rates [50]. A rapid decline in proteinuria indicates effective control of glomerular inflammation and immune complex deposition. Multiple studies, such as the TESTING trial, show that decreased proteinuria is associated with a slower decline in renal function, making it a primary endpoint in clinical trials recognized by both the FDA (Food and Drug Administration) and the EMA (European Medicines Agency). The significance of the eGFR slope as a long-term outcome measure lies in its role as a direct indicator of kidney function decline. The eGFR slope reflects long-term changes in glomerular filtration rate and serves as a key predictor of whether the disease has progressed to end-stage renal disease (ESRD). Although a reduction in proteinuria may suggest stable eGFR, some patients may experience a “proteinuria-eGFR disconnect,” where glomerular function (eGFR) appears normal despite significant protein leakage in the urine (proteinuria). Therefore, the eGFR slope is a vital marker for confirming whether treatment effectively preserves renal function. Nevertheless, a comprehensive assessment of long-term renal protection should also include histological improvements and biomarkers, such as Gd-IgA1 levels. In the short term, focus should be on monitoring the reduction in proteinuria, as this serves as an early indicator of treatment effectiveness. However, long-term management requires ongoing assessment of the eGFR slope to ensure that treatment not only relieves symptoms but also significantly slows the progression of IgA nephropathy.

Based on currently limited evidence, we cautiously conclude that new biologic treatments targeting BAFF/APRIL may reduce the 24-h urine protein-to-creatinine ratio (UPCR) and circulating Gd-IgA_1_ levels in patients with IgAN, while potentially slowing the decline in estimated glomerular filtration rate (eGFR). It is noteworthy that BAFF/APRIL dual antagonists might lower serum levels of IgG, IgA, and IgM, potentially impairing patients’ immune defenses. New biologic treatments targeting BAFF/APRIL are in Phase III trials. We must continue monitoring their safety to confirm they are safe and effective.

Regarding the placement of BAFF/APRIL-targeted therapies in current clinical practice, we believe that, before approval of Phase III data, they may be used as a priority option in clinical trials or off-label (based on Phase II evidence) for high-risk patients who have failed standard treatment. If Phase III data prove favorable, this could become a novel therapeutic option for high-risk progressive IgA nephropathy, especially for patients intolerant to corticosteroids or seeking safer targeted therapies. However, its final positioning will depend on evidence of long-term kidney protection, safety data, pricing, and healthcare reimbursement policies. If successful, this could transform the treatment approach for IgA nephropathy, marking an important shift from supportive care to targeted immunomodulation.

## 7. Conclusions

Based on current research progress, BAFF/APRIL biologics demonstrate breakthrough potential in the treatment of IgAN; however, larger-scale clinical trials and laboratory studies are still needed to demonstrate the long-term efficacy and safety of BAFF/APRIL biologics in the treatment of IgAN. Despite these challenges, targeted intervention in the BAFF/APRIL system holds promise to reshape the therapeutic landscape for IgAN, particularly for patients unresponsive to conventional treatments [14,131]. In recent years, the discovery of several new biomarkers has opened up new possibilities for personalized medicine in IgAN. These include Gd-IgA1 and its antibodies, components of the complement system (such as C4d and FHR-1), urinary protein markers (such as MMP-7 and KIM-1), and microRNAs (such as miR-148b and let-7b), which show promise for both diagnosis and prognosis assessment [132,133]. Therefore, the future management of IgA nephropathy could benefit from enhanced comprehensive assessment of multidimensional biomarkers. This includes developing a risk stratification model that integrates clinical indicators (e.g., proteinuria, eGFR), pathological scores (MEST-C), and molecular biomarkers (e.g., Gd-IgA1, miRNA, complement). Additionally, it is important to develop therapeutic response and prognostic assessments tailored to different treatments (e.g., corticosteroids, targeted therapies). Standardizing detection methods and broadening sample diversity can promote the clinical translation and universal validation of biomarkers, ultimately shifting from a one-size-fits-all approach to precision, stratified diagnostic and therapeutic strategies.

## 8. Literature Search

This manuscript is a non-systematic narrative review. This study mainly conducted searches in the PubMed database, Google Scholar, and ClinicalTrials.gov. The references include publications from 2002 to 2025, with more focus on those published between 2015 and 2025.

## Data Availability

No new data were created or analyzed in this study.
